# Early-life undernutrition increases the risk of death from chronic diseases in adulthood: a population-based cohort study

**DOI:** 10.1186/s41256-025-00422-0

**Published:** 2025-07-10

**Authors:** Mengqiu Wu, Hongrui Tian, Chuanhai Guo, Zhen Liu, Yaqi Pan, Fangfang Liu, Ying Liu, Wenlei Yang, Huanyu Chen, Zhe Hu, Mengfei Liu, Zhonghu He, Yang Ke

**Affiliations:** 1 Key Laboratory of Carcinogenesis and Translational Research (Ministry of Education/Beijing), Department of Genetics, Peking University Cancer Hospital and Institute, #52 Fucheng Rd, Beijing, 100142 China; 2 State Key Laboratory of Molecular Oncology, Beijing Key Laboratory of Carcinogenesis and Translational Research, Department of Genetics, Peking University Cancer Hospital and Institute, #52 Fucheng Rd, Beijing, 100142 China

**Keywords:** Early-life undernutrition, Chronic non-communicable diseases, Chinese great famine, Mortality

## Abstract

**Background:**

Early-life undernutrition, particularly during critical developmental periods, may have lasting impacts on non-communicable diseases (NCDs) in adulthood. The Chinese Great Famine (1959–1961) provides a unique opportunity to evaluate these effects in a large-scale population study. To investigate the impact of early-life undernutrition on adult mortality due to NCDs in individuals exposed to the Chinese Great Famine.

**Methods:**

We analyzed data from a medical insurance database in Hua County, China, including 15,088 individuals born during the famine (1959–1961) and 49,924 individuals deemed unexposed because they were born after the famine (1962–1964), with follow-up from 2012 to 2023. Multivariable Cox regression and competing risks regression were used to assess the association between early-life undernutrition and mortality.

**Results:**

Early-life undernutrition was associated with increased risks of all-cause mortality (HR_adjusted_ = 1.49, 95% CI 1.37–1.62), cancer mortality (HR_adjusted_ = 1.41, 95% CI 1.22–1.64), cardiovascular and cerebrovascular diseases mortality (HR_adjusted_ = 1.51, 95% CI 1.34–1.71), and chronic obstructive pulmonary disease mortality (HR_adjusted_ = 4.37, 95% CI 2.51–7.61). Subgroup analysis revealed that the exposed group had a higher risk of death from lung, esophageal, gastric, hepato-biliary, and pancreatic cancers, cerebrovascular disease and cardiovascular disease.

**Conclusions:**

This study demonstrates the long-term adverse effects of early-life undernutrition on NCD mortality in adulthood, underscoring the importance of nutritional interventions during critical developmental periods to reduce the burden of NCDs.

***Clinical trial registration*:**

Endoscopic Screening for Esophageal Cancer in China (ESECC) randomized controlled trial (Clinical trial: NCT01688908).

**Supplementary Information:**

The online version contains supplementary material available at 10.1186/s41256-025-00422-0.

## Introduction

Non-communicable diseases (NCDs) have been rising as a significant global public health challenge, featured by their prolonged course and complicated etiology [1]. According to the Global Burden of Disease (GBD) data in 2021, approximately 46 million deaths were attributable to NCDs globally, among which about 29 million were caused by three major chronic diseases: cardiovascular and cerebrovascular diseases, cancer, and chronic obstructive pulmonary disease, ranked in descending order of disease burden 2.

The onset and progression of NCDs are a complex process influenced by multiple factors, including genetic predisposition, lifestyle behaviors, socioeconomic backgrounds, and psychological factors. Early life is a critical period during which rapid growth and development of the organs occur, and the metabolic pattern of the body is established, exerting long-term and profound effects on an individual’s risk and outcomes of NCDs [3]. David Barker first proposed the “Fetal Origins of Adult Disease (FOAD)” hypothesis stemming from the association between late-onset coronary heart disease and low birth weight he and his colleagues reported in 1986 [4], revealing the non-negligible impact of inadequate fetal nutrition on subsequent NCD development in adult life [5, 6]. This hypothesis was further updated as the “Developmental Origins of Health and Disease (DOHaD)” theory, which links the risk of adult chronic diseases with environmental factors during early developmental stages, underscoring a potential transgenerational effect [1].

To investigate the impact of early-life undernutrition on NCDs in adulthood, the well-accepted optimal study design is a birth cohort with long-term follow-up. However, the measurement of individual-level early-life nutritional status in a huge population, especially in infants, poses significant challenges in real-world practices. As an alternative strategy addressing this problem, famines affecting a known geographic area during a known time period were commonly taken as a “natural experiment” to establish cohorts suffering vs. not suffering from early-life undernutrition. To date, several cohort studies in famine-affected areas have shown that early-life undernutrition increases the risk of all-cause and NCD mortality in adulthood [7–11]. However, those studies were limited by small sample sizes (< 10,000), inadequate population representativeness, insufficient control for confounding factors, and mostly restricted to a single NCD.

In this study, we conducted a whole population cohort study consisting of individuals born between 1959 and 1964 (3 years during the famine vs. 3 years after the famine) in an area affected by the 1959–1961 Chinese Great Famine [12]. These individuals were then followed up during 2012–2023 to determine the potential associations between early-life undernutrition and the risk of all-cause mortality, and mortality from three major NCDs in adulthood: including cancer, cardiovascular and cerebrovascular diseases, and chronic obstructive pulmonary disease. We also performed individualized covariate adjustment using epidemiological survey data from large-scale population-based cohorts in the same study area to control potential confounding effects. To comprehensively assess the association between early-life undernutrition and chronic diseases mortality risk in adulthood, this study employs a dual-data validation strategy: (1) whole population analysis based on the healthcare population registry system, and (2) individualized risk assessment utilizing epidemiological survey cohorts. Through synergistic integration of these complementary data sources from different dimensions, we aim to collaboratively validate the research hypotheses.

## Methods

### Study area

The study area, Hua County, is located in Henan Province in northern China. In 2012, the county had a permanent resident population of 1.24 million, which declined to 1.15 million in 2023. In 2023, the county’s per capita Gross Domestic Product was 22,265.9 Chinese Yuan (US $3,063.79) [13]. Hua County was devastated by the “Chinese Great Famine” in 1959–1961. Official population data indicated that the mortality in Hua County reached 39.6‰ in 1960, doubling the average level from 1956 to 1958 [12, 14]. The county has a stable population (annual variation rate < 1%) and low mobility (out-migration rate < 5% between 2012 and 2023), making it an ideal location for long-term follow-up studies. Furthermore, 90% of the residents are engaged in agriculture in Hua County, making it a representative area for studying the impact of famine. Therefore, this study defines Hua County as a primary famine-affected area and considers its population as the study cohort.

### Study subjects

**Study subjects in whole population analysis.** In this study, we retrospectively extracted the registry information, including personal ID, sex, date of birth and residence (detailed to the level of village), of local residents from the New Rural Co-operative Medical Scheme (NRCMS) in 2012 in Hua County, which is the sole government-run health insurance system for rural residents in China, achieving a coverage of > 95% in the study area at 2012 [15]. The records extracted from the NRCMS were then linked with the death registry data of Hua County.

The individuals meeting the following inclusion criteria were included: (1) being born within the period from 1 January 1959 to 31 December 1964; (2) having complete personal ID and sex information; and (3) being alive as of 1 January 2012 according to the local death registry. A total of 1,234,851 individuals were initially included in the whole population analysis. As shown in the flow diagram (see Supporting Material), 1,169,593 individuals born outside the period from 1 January 1959 to 31 December 1964 were excluded, leaving 65,258 individuals eligible based on the inclusion criteria. Among these, 122 individuals were further excluded due to missing or incorrect information, including 31 with invalid personal IDs and 91 with errors in names, villages, or sex. This resulted in 65,136 individuals with complete personal ID and sex information. Additionally, 124 individuals with recorded death dates prior to cohort enrollment on 1 January 2012 were excluded, leaving a final cohort of 65,012 participants.

**Study subjects in individual-level analysis of population-based cohorts.** In order to better control the potential confounding effects of age at enrollment and other risk factors on the association between the famine exposure and adulthood NCD outcomes, individualized data from two representative population-based cohorts in Hua County were used, namely the Endoscopic Screening for Esophageal Cancer in China (ESECC) trial [16–18] and Anyang Esophageal Cancer Cohort Study (AECCS) [19] conducted by our research team. Both studies were designed as community-based screening cohorts for upper gastrointestinal cancers and collected individual-level information using questionnaire survey by trained researchers according to a standard operation procedure, enabling adjustment for individual-level confounders (see supplementary methods for details). Both the ESECC and AECCS cohorts collected participants’ personal ID, which function as the unique identifiers in the NRCMS system. This allowed us to accurately link individual-level covariates with NRCMS reimbursement records and death registry data. The inclusion criteria for participants in the population-based cohorts are identical to those for the whole population analysis.

### Exposure definition

Early-life undernutrition exposure was defined based on participants’ date of birth. Individuals born between 1 January 1959 and 31 December 1961, i.e., who were under the age of three during the 1959–1961 Chinese Great Famine, were classified as having been exposed to early-life undernutrition. The unexposed participants were those born between 1 January 1962 and 31 December 1964.

### Outcomes ascertainment and follow-up

The primary outcomes of this study include all-cause mortality, cancer mortality, cardiovascular and cerebrovascular diseases mortality, and chronic obstructive pulmonary disease mortality during the follow-up period. Secondary outcomes involve mortality from lung cancer, esophageal cancer, gastric cancer, colorectal cancer, hepato-biliary cancer, pancreatic cancer, cardiovascular disease, and cerebrovascular disease separately.

Death events were ascertained using the death registry data from the Centers for Disease Control and Prevention (CDC) in Hua County. The registry records, after de-identification, include the date of death, the primary cause of death (coded according to the ICD-10), and basic personal information (e.g., personal ID, sex, date of birth, and residence). Linkage between the death registry, the NRCMS, and the individual-level databases of the ESECC and AECCS cohorts was performed using the encrypted personal ID as the primary matching variable. For records lacking a valid personal ID, we employed a fuzzy matching algorithm based on name, sex, date of birth, and residence (town and village) to identify death events. Any discrepancies were manually verified through double data entry and logical consistency checks, ensuring the accuracy of the linkage [20].

For the whole population analysis, the start of follow-up was set at 1 January 2012. For the individualized analysis of population-based cohorts, follow-up for ESECC started from the actual date of entry into the cohort for each individual, which occurred during 2012–2016; for the AECCS, although the cohort enrollment occurred during 2006–2009, to ensure a follow-up duration and baseline risk of death comparable with those of ESECC, the start of follow-up for this study was set to 1 January 2012. The end date of follow-up was set at 31 December 2023 for all analyses. Time at risk was calculated from the enrollment date until the end of follow-up or the date of death, whichever occurred first.

This study maintained a high quality of follow-up using linkage with the NRCMS database and the death registry from Hua County CDC. No loss to follow-up was observed during the study period due to the comprehensive coverage and robust data linkage mechanisms.

### Statistical analysis

**Whole population analysis.** Sex distribution was compared between exposed and unexposed cohorts using the Chi-square test, and age and follow-up duration compared using Wilcoxon rank sum test. Survival curves for both exposed and unexposed cohorts were estimated using Kaplan–Meier method and compared using log-rank test. Associations between early-life undernutrition and all-cause mortality were evaluated using multivariable Cox proportional hazards model adjusted for sex. For all the other outcome events, competing risk regression models were employed treating causes of death other than the cause of interest as competing events. The competing risk model can estimate the cumulative incidence rate and hazard ratio of the target event more accurately by taking into account the influence of competing events.

#### Individualized analysis of population-based cohorts

**Individualized analysis of population-based cohorts.** Based on individual-level questionnaire data from two population-based cohorts conducted in Hua County, we further constructed three multivariable Cox models for all-cause mortality and three competing risk models for cause-specific mortality (labelled as Models 0, 1, 2, and 3) that adjusted for well-established determinants of death. For each outcome, Model 0 adjusted for sex; Model 1 adjusted for age at enrollment, sex, education (illiterate, primary school, junior high school, or high school and above), occupation (non-laborer or laborer), and marital status (married or others); Model 2 further included cigarette smoking (yes, no) and alcohol drinking (yes, no); and Model 3 further adjusted for body mass index (BMI; < 18.5 kg/m^2^, 18.5–24.9 kg/m^2^, 25.0–29.9 kg/m^2^, or ≥ 30 kg/m^2^), and additionally incorporated cancer family history for cancer mortality.

All statistical analyses were performed using STATA (Version 15.0; Stata Corp LLC, TX, USA). All tests were two-tailed, and *P* values < 0.05 were considered statistically significant.

### Patient and public involvement statement

Patients and the public participated in individualized data collection for the Endoscopic Screening for Esophageal Cancer in China (ESECC) trial and the Anyang Esophageal Cancer Cohort Study (AECCS). They provided demographic, lifestyle, and health information through questionnaires and interviews. Research questions and survey tools were refined based on input from participants and community health workers. Participation burden was minimized through informed consent and consultation with local healthcare providers.

Findings will be shared with the community via health education programs, public health recommendations, and local events.

## Results

### Whole population analysis

The whole population cohort enrolled 65,012 individuals, including 15,088 famine exposed participants (born between 1 January 1959 and 31 December 1961) and 49,924 unexposed participants (born between 1 January 1962 and 31 December 1964). The median age in the exposed cohort was three years older than in the non-exposed cohort. Sex was well balanced between these two cohorts (Table 1). The median follow-up duration for both the exposed and unexposed cohorts were 12.00 years.

**Table 1:** Distribution of age at enrollment, sex and follow-up duration in early-life undernutrition exposed and unexposed cohorts in Hua County, China, 2012–2023

Variable	Total (n = 65,012)		Undernutrition exposed cohort^a^ (n = 15,088)		Unexposed cohort^b^ (n = 49,924)	*P* value^d^
	n (%)		n (%)		n (%)	
Age at enrollment^c^ (y)						
Median (interquartile range)	49 (48, 50)		52 (51, 53)		49 (48, 49)	< 0.001
Sex						
Female	33,514 (51.55)		7788 (51.62)		25,726 (51.53)	0.852
Male	31,498 (48.45)		7300 (48.38)		24,198 (48.47)	
Follow-up duration^c^ (y)						
Median (interquartile range)	12.00 (12.00,12.00)		12.00 (12.00,12.00)		12.00 (12.00,12.00)	NA^e^

^a^ The early-life undernutrition exposed cohort consisted of residents in the Hua County born between 1 January 1959 and 31 December 1961, who were followed up as of 1 January 2012

^b^ The unexposed cohort consisted of residents in the Hua County born between 1 January 1962 and 31 December 1964, who were followed up as of 1 January 2012

^c^ The follow-up duration is set as from 1 January 2012 up to 31 December 2023, or the date of death

^d^ *P* values were generated from the Chi-square test and Wilcoxon rank sum test

^e^ Not applicable because the follow-up duration for subjects alive at the end date was manually set as 12 years

**Fig. 1:**
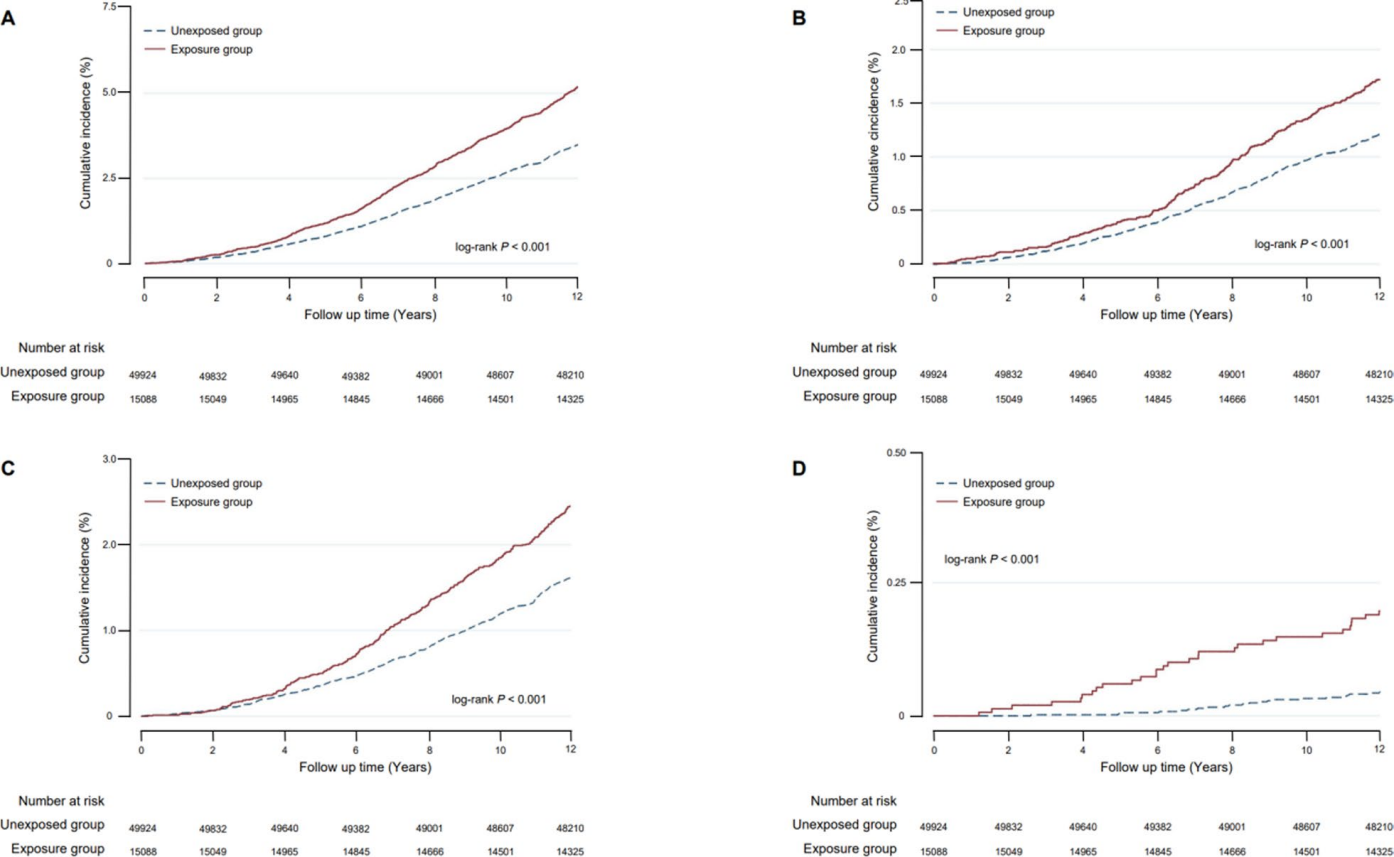
Cumulative incidence curves of all-cause death (**A**), cancer death (**B**), cardiovascular and cerebrovascular diseases death (**C**), and chronic obstructive pulmonary disease death (**D**) in early-life undernutrition exposed and unexposed cohorts in Hua County, China, 2012–2023. *HR* hazard ratio, *CI* confidence interval

As shown in Table 2, the all-cause mortality rate in the early-life undernutrition exposed cohort was 4.30 per 1000 person years (PY) (95% CI 4.00–4.62), significantly higher than that in the unexposed cohort (2.90 per 1000 PY; 95% CI 2.77–3.04; *P* < 1.00 × 10^–16^). Significantly higher mortality rates of cancer, cardiovascular and cerebrovascular diseases, and chronic obstructive pulmonary disease, were also observed in the famine exposed group as compared to the unexposed cohort (Table 2). Survival analysis revealed a similar pattern (Fig. 1).

**Table 2:** The mortality rate and cumulative mortality of all-cause death and chronic disease death in early-life undernutrition exposed and unexposed cohorts in Hua County, China, 2012–2023

Outcome events	Undernutrition exposed cohort^a^ (n = 15,088)			Unexposed cohort^b^ (n = 49,924)			*P* value^c^
	Number of deaths	Mortality rate (per 1000 person-years)	Cumulative mortality (%)	Number of deaths	Mortality rate (per 1000 person-years)	Cumulative mortality (%)	
All-cause death	763	4.30 (4.00–4.62)	5.06 (4.71–5.42)	1716	2.90 (2.77–3.04)	3.44 (3.28–3.60)	< 1.00 × 10^–16^
Cancer death	253	1.43 (1.26–1.61)	1.68 (1.48–1.89)	594	1.00 (0.93–1.09)	1.19 (1.10–1.29)	3.20 × 10^–6^
Cardiovascular and cerebrovascular diseases death	360	2.03 (1.83–2.25)	2.39 (2.15–2.64)	792	1.34 (1.25–1.44)	1.59 (1.48–1.70)	6.83 × 10^–11^
Chronic obstructive pulmonary disease death	29	0.16 (0.11–0.24)	0.19 (0.13–0.28)	22	0.04 (0.02–0.06)	0.04 (0.03–0.07)	1.67 × 10^–7^

^a^ The early-life undernutrition exposed cohort consisted of residents in the Hua County born between 1 January 1959 and 31 December 1961, who were followed up as of 1 January 2012

^b^ The early-life undernutrition unexposed cohort consisted of residents in the Hua County born between 1 January 1962 and 31 December 1964, who were followed up as of 1 January 2012

^c^ *P* values were derived from Poisson regression

To further assess the effect of 3 year age gap between groups on the observed effect of famine exposure, we calculated mortality rates of the famine exposed and non-exposed individuals based on overlapping person-years within the 51–62 age-group during follow-up. We still observed significantly higher mortality rates in the famine exposed subjects after control for age (Supplementary Table 1).

**Fig. 2:**
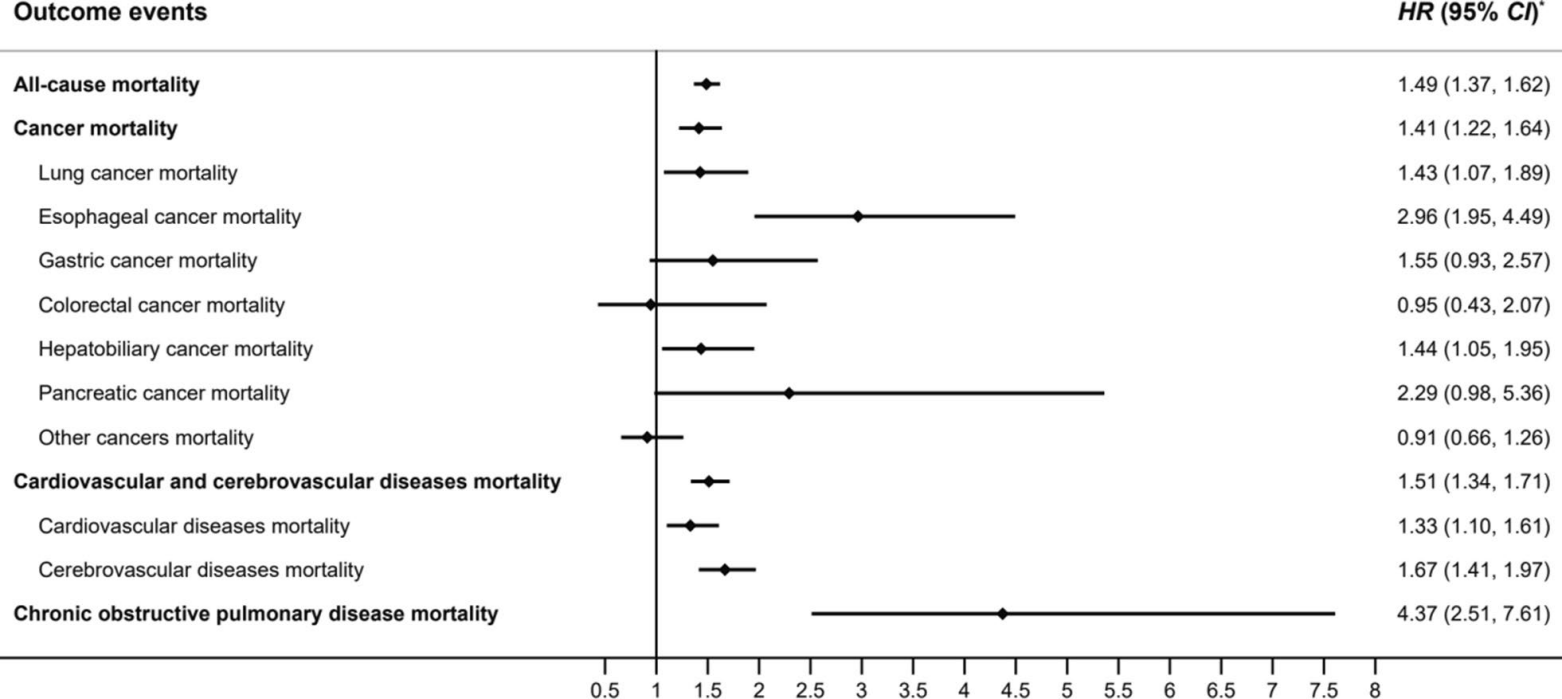
The effect of early-life undernutrition exposure on death from NCDs in late adulthood, among 65,012 residents in Hua County, 2012–2023. ^*^ The HRs were adjusted for sex. ^*^All-cause mortality was analyzed using Cox proportional hazards models, while cancer, cardiovascular and cerebrovascular diseases, and chronic obstructive pulmonary disease mortality were assessed using competing risks regression models

As shown in Fig. 2, early-life undernutrition increased the risk of all-cause mortality (HR_adjusted_ = 1.49, 95% CI 1.37–1.62), cancer mortality (HR_adjusted_ = 1.41, 95% CI 1.22–1.64), cardiovascular and cerebrovascular diseases mortality (HR_adjusted_ = 1.51, 95% CI 1.34–1.71), and chronic obstructive pulmonary disease mortality (HR_adjusted_ = 4.37, 95% CI 2.51–7.61) after adjusting for sex.

Subgroup analysis stratified by cancer type revealed that the exposed group had a higher risk of death from lung cancer (HR_adjusted_ = 1.43, 95% CI 1.07–1.89), esophageal cancer (HR_adjusted_ = 2.96, 95% CI 1.95–4.49), gastric cancer (HR_adjusted_ = 1.55, 95% CI 0.93–2.57), hepato-biliary cancer (HR_adjusted_ = 1.44, 95% CI 1.05–1.95), and pancreatic cancer (HR_adjusted_ = 2.29, 95% CI 0.98–5.36) (Fig. 2 and Supplemental Table 2). For cardiovascular and cerebrovascular diseases, early-life undernutrition had a differential impact on mortality. Subgroup analysis stratified by cardiovascular and cerebrovascular diseases revealed that the exposed group had a higher risk of death from cerebrovascular disease mortality (HR_adjusted_ = 1.67, 95% CI 1.41–1.97) and cardiovascular disease mortality (HR_adjusted_ = 1.33, 95% CI 1.10–1.61) (Fig. 2).

### Individualized analysis based on population cohorts

To further adjust for potential risk factors of NCDs-related death, including age at enrollment, sex, education, occupation, and marital status, cigarette smoking, alcohol drinking and BMI, we conducted individualized analysis based on two representative cohort studies, comprising 1948 individuals exposed to famine and 6189 individuals not exposed to famine. The distribution of covariates between famine-exposed and unexposed groups is detailed in Supplemental Table 3. The exposed group had a higher proportion of older individuals, lower educational attainment, lower BMI, and a higher prevalence of cancer family history compared to the unexposed group. Similar to the whole population analysis, significantly higher rates of all-cause mortality and death from cancer, cardiovascular and cerebrovascular diseases were also observed in the famine exposed group as compared to the unexposed cohort (Supplemental Table 4 and Supplemental Fig. 1).

**Fig. 3:**
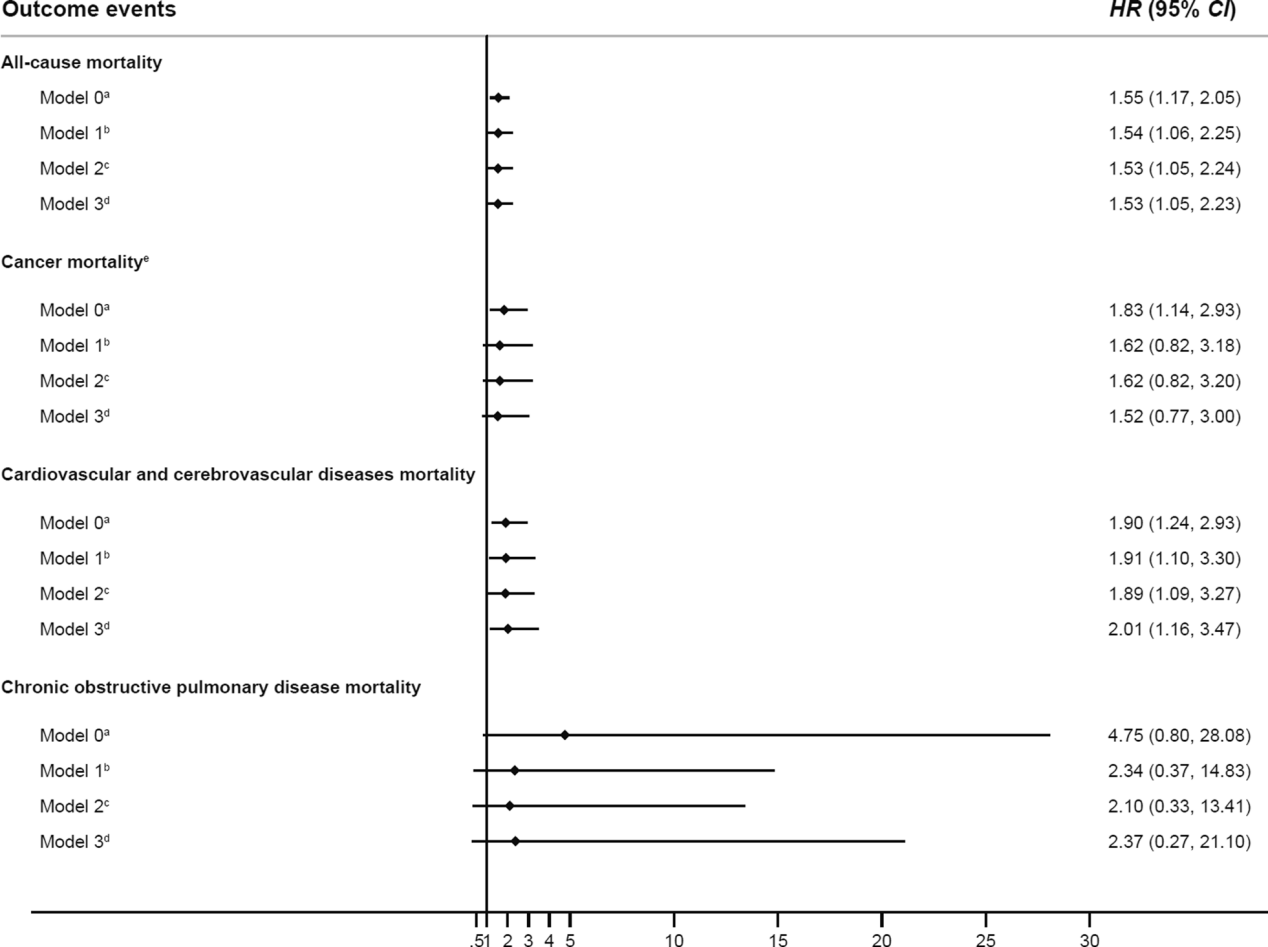
The effect of early-life undernutrition exposure on mortality estimated using multivariable models, among 8137 subjects with individual-level covariate information from the ESECC trial and AECCS in Hua County, 2012–2023. ^a^ The HRs from Model 0 were adjusted for sex. ^b^ The HRs from Model 1 were adjusted for age at enrollment, sex, education, occupation, and marital status. ^c^ The HRs from Model 2 were adjusted for age at enrollment, sex, education, occupation, marital status, cigarette smoking and alcohol drinking. ^d^ The HRs from Model 3 were adjusted for age at enrollment, sex, education, occupation, marital status, cigarette smoking, alcohol drinking and body mass index (BMI). ^e^ Cancer family history was added to all the models for cancer mortality. ^*^All-cause mortality was analyzed using Cox proportional hazards models, while cancer, cardiovascular and cerebrovascular diseases, and chronic obstructive pulmonary disease mortality were assessed using competing risks regression models

After full adjustment of potential confounders using individual-level epidemiological data, we observed results consistent with those from the whole population analysis. Specifically, early-life undernutrition exposed individuals exhibited increased hazards of all-cause mortality (HR_model 3_ = 1.53, 95% CI 1.05–2.23), cancer mortality (HR_model 3_ = 1.52, 95% CI 0.77–3.00), cardiovascular and cerebrovascular disease mortality (HR_model 3_ = 2.01, 95% CI 1.16–3.47) and chronic obstructive pulmonary disease mortality (HR_model 3_ = 2.37, 95% CI 0.27–21.10), compared to the unexposed individuals (Fig. 3).

## Discussion

Preventing and treating NCDs are pivotal for health promotion, as highlighted by the World Health Organization’s report that over 70% of deaths are attributable to chronic conditions [2]. Besides early diagnosis strategies such as screening, primary prevention targeting etiological factors assumes equally, if not more, critical importance. This study reveals that early-life undernutrition significantly elevates the risk of mortality from various NCDs in late adulthood, including cancer, cardiovascular and cerebrovascular diseases, and chronic obstructive pulmonary disease, affecting respiratory, cardiovascular, and digestive systems. Our findings underscore the imperative of ensuring adequate nutrition for infants and toddlers (up to age three) to avoid the risk of NCDs in adulthood, thereby reducing the loss of healthy life expectancy and premature death [21, 22].

The impact of early-life nutritional deficiencies may vary across organs and diseases. However, there are few studies investigating the effect of early-life undernutrition on the risk of mortality from several different diseases in one defined population. In this study, we found that early-life undernutrition had a widespread impact on gastrointestinal cancer, cardiovascular and cerebrovascular diseases, and chronic obstructive pulmonary disease, indicating its impact on the respiratory, circulatory, and digestive systems. However, there is a considerable variability in the magnitude of the effect. For instance, the effect was more pronounced for esophageal cancer, gastric cancer, pancreatic cancer, lung cancer, and hepatobiliary cancer, whereas negligible for colorectal cancer. The mechanisms underlying this heterogeneity is yet to be elucidated in future researches. For cardiovascular and cerebrovascular diseases, the unfavorable impact of early-life undernutrition was more evident on cerebral vessels as compared to that on the coronary arteries, suggesting that the period before three years in age may be particularly critical for cerebrovascular development.

The potential mechanisms linking early-life exposure to undernutrition and NCDs mortality in adulthood may be complicated. First, undernutrition during fetal and infant period may trigger thriftiness mechanisms affecting the growth of body organs differentially, potentially providing selective protection for brain growth. Such adaptive responses may permanently alter body structure and function, ensuring early survival but potentially increasing the risk of NCDs mortality in adulthood [23]. For example, animal experiments have revealed associations between early-life undernutrition and arterial wall remodeling, late-life arterial stiffness, and hypertension [24]. Similarly in humans, individuals who experienced early-life undernutrition are more likely to develop hypertension and hyperglycemia, which are well-known risk factors for cardiovascular diseases [25]. Second, deficiencies in micronutrients such as iron, folate, and vitamins due to insufficient consumption during fetal and early life stages may impair growth and metabolic functions in infants and young children [26, 27]. Several studies suggest that deficiencies in vitamins A and D during early-life may impact lung development with mechanisms largely unknown [8, 28]. It is speculated that reduced nutrition or oxygen supply may damage cellular and molecular activities in the lungs, leading to structural and functional changes, which in turn raise the long-term risk of lung cancer and chronic obstructive pulmonary disease mortality in adulthood [29]. Notably, while we observed a markedly elevated hazard ratio (HR = 4.37, 95% CI 2.51–7.61) for chronic obstructive pulmonary disease mortality in the whole-population analysis, the width of the confidence interval reflects limited statistical precision. This is consistent with the broad confidence intervals observed in individual-level analyses, suggesting that some associations may be imprecise in the current study. Prospective studies that can capture more COPD death events are needed to further validate these findings. Third, physiological stress and metabolic adaptations induced by starvation and undernutrition may persistently affect metabolism and endocrine function, thereby increasing the risk of NCDs mortality in later life [26].

As mentioned above, several studies have investigated the association between early-life undernutrition and NCDs mortality in adulthood [7–11, 30–32], including those based on the Chinese Great Famine [33, 34]. However, no conclusive findings have been generated regarding the significance, value, and related policies of early-life nutritional support to date. This may be attributable to limitations in existing research, including small sample sizes, suboptimal study design, and insufficient control for key confounders such as lifestyle factors (e.g., smoking, alcohol consumption) and metabolic indicators (e.g., BMI trajectory). Notably, no study has simultaneously analyzed multi-organ system outcomes in a unified analytical framework.

Compared to the previous studies, our research has several advantages. First, we used data from a population registry that covers the whole population of populous province with ~ 1.2 million people. Applying strict inclusion and exclusion criteria, we included 15,088 individuals in the exposed group and 49,924 in the unexposed group, and followed this cohort for 12 years. This study design ensures an ideal population representativeness and sufficient statistical power to detect associations between early-life undernutrition and NCD deaths in a robust manner. Second, this study examines the effect of early-life undernutrition exposure on various chronic diseases, including cancer, cardiovascular and cerebrovascular diseases, and chronic obstructive pulmonary disease, which in combination contribute to over 70% of NCDs mortality burden. The findings of this study may therefore help to generate a comprehensive view regarding the harm of early-life nutrition deficiency in adulthood. Third, this study leverages two community-based cohorts in Hua County, which provided individual-level risk factor information and allowed control for covariates, especially for the age difference of individuals between groups, to minimize potential confounding bias toward mortality. This validation analysis indicated that the association between early-life undernutrition and NCD risk in adulthood remained significant after carefully adjustment for other death determinates, supporting the validity of conclusions from our whole population analysis.

Several limitations should also be noted in this study. First, although covering the whole population of the study area, it is a single-center study. Verification of the findings in other populations is needed. Second, since the study cohort only included individuals alive as of January 1, 2012, those who died during the famine due to undernutrition or in early adulthood were excluded, potentially leading to an underestimation of the long-term impacts of early-life undernutrition. Third, due to the unavailability of more detailed information regarding the beginning and ending time of the Great Famine in China, misclassification of the famine exposed and non-exposed individuals would be inevitable based on calendar years. Fourth, this study did not consider the direct impact of the famine on the parents of children born between 1 January 1962 and 31 December 1964, including potential epigenetic effects and other intergenerational influences. This limitation may lead to an underestimation of the association effects. However, this does not affect the conclusions of this study. Finally, this study lacks granular dietary data from both childhood and adulthood. This precluded assessment of potential interactions between early-life undernutrition and subsequent nutritional patterns, which may modulate chronic disease trajectories.

## Conclusion

Our study reveals that early-life undernutrition increase the risk of death from all causes, and from NCDs including cancer, cardiovascular and cerebrovascular diseases, and chronic obstructive pulmonary disease in late adulthood. This finding underscores the importance of paying special attention to the nutritional needs of infants and toddlers to prevent NCDs in adulthood, thereby reducing the loss of healthy life years.

## Supplementary Information

Additional file 1.

## Data Availability

The datasets used and/or analyzed during the current study are not publicly available due to privacy concerns of the participants but are available from the corresponding author upon reasonable request.

## Abbreviations

AECCS: Anyang esophageal cancer cohort study
CDC: Center for disease control and prevention
DOHaD: Developmental origins of health and disease
ESECC: Endoscopic screening for esophageal cancer in China
FOAD: Fetal origins of adult disease
GBD: Global burden of disease
NCDs: Non-communicable diseases
NRCMS: New rural co-operative medical scheme
