# Supplementary material for: Early-life undernutrition increases the risk of death from chronic diseases in adulthood: a population-based cohort study
Source: Glob Health Res Policy. 2025 Jul 10;10:28. doi: 10.1186/s41256-025-00422-0 (PMC12243173; doi:10.1186/s41256-025-00422-0)

**Supplementary Material**

**Content**

[Supplementary table and figure 2](#_Toc179986108)

[Supplemental table 1 2](#_Toc179986109)

[Supplemental table 2 3](#_Toc179986110)

[Supplemental table 3 4](#_Toc179986111)

[Supplemental table 4 5](#_Toc179986112)

[Supplemental figure 1 6](#_Toc179986113)

[Supplementary methods 6](#_Toc179986115)

[Introduction to the ESECC trial 6](#_Toc179986116)

[Introduction to the AECCS study 7](#_Toc179986117)

[Reference 8](#_Toc179986118)

**Supplementary table and figure**

**Supplemental table 1.**The mortality rate of all-cause death and chronic disease death in early-life undernutrition exposed and unexposed cohorts based on overlapping person-years (aged 51-62) during follow-up from 2012 to 2023.

| **Outcome events** | **Undernutrition exposed cohort^a^** | |  | **Unexposed cohort^b^** | | ***RR* (95% *CI*)** | ***P* value^c^** |
| --- | --- | --- | --- | --- | --- | --- | --- |
|  | **Number of deaths** | **Mortality rate (per 1000 person-years)** |  | **Number of deaths** | **Mortality rate (per 1000 person-years)** |  |  |
| All-cause death | 573 | 4.30 (4.00-4.62) |  | 1681 | 2.90 (2.77-3.04) | 1.24 (1.13-1.36) | <1.00×10^-5^ |
| Cancer death | 192 | 1.43 (1.26-1.61) |  | 586 | 1.00 (0.93-1.09) | 1.19 (1.01-1.40) | 0.036 |
| Cardiovascular and cerebrovascular diseases death | 265 | 2.03 (1.83-2.25) |  | 773 | 1.34 (1.25-1.44) | 1.25 (1.08-1.43) | 0.002 |
| Chronic obstructive pulmonary disease death | 21 | 0.16 (0.11-0.24) |  | 22 | 0.04 (0.02-0.06) | 3.47 (1.91-6.31) | 4.55×10^-5^ |

^a^ The early-life undernutrition exposed cohort consisted of residents in the Hua County born between 1 January 1959 and 31 December 1961, who were followed up as of 1 January 2012.

^b^ The early-life undernutrition unexposed cohort consisted of residents in the Hua County born between 1 January 1962 and 31 December 1964, who were followed up as of 1 January 2012.

^c^ *P* values were derived from Poisson regression.

**Supplemental table 2.** Subgroup analysis regarding the effect of early-life undernutrition exposure on mortality from various NCDs separately in adulthood derived from multivariable competing risk regression models, among 65012 subjects in Hua County, 2012-2023.

| **Outcome events** | **Early-life undernutrition Exposure** | | ***HR* (95% *CI*)** |
| --- | --- | --- | --- |
|  | **No** | **Yes** |  |
| Lung cancer death |  |  |  |
| No | 49766 | 15020 | Ref |
| Yes | 158 | 68 | 1.43 (1.07-1.89) |
| Esophageal cancer death |  |  |  |
| No | 49877 | 15046 | Ref |
| Yes | 47 | 42 | 2.96 (1.95-4.49) |
| Gastric cancer death |  |  |  |
| No | 49877 | 15066 | Ref |
| Yes | 47 | 22 | 1.55 (0.93-2.57) |
| Colorectal cancer death |  |  |  |
| No | 49896 | 15080 | Ref |
| Yes | 28 | 8 | 0.95 (0.43-2.07) |
| Hepatobiliary cancer death |  |  |  |
| No | 49790 | 15030 | Ref |
| Yes | 134 | 58 | 1.44 (1.05-1.95) |
| Pancreatic cancer death |  |  |  |
| No | 49911 | 15079 | Ref |
| Yes | 13 | 9 | 2.29 (0.98-5.36) |
| Cardiovascular diseases death |  |  |  |
| No | 49550 | 14938 | Ref |
| Yes | 374 | 150 | 1.33 (1.10-1.61) |
| Cerebrovascular diseases death |  |  |  |
| No | 49506 | 14878 | Ref |
| Yes | 418 | 210 | 1.67 (1.41-1.97) |

^*^ The HRs were adjusted for sex.

^*^Subgroup analyses were assessed using competing risks regression models.

**Supplemental table 3.** The distribution of demographic characteristics and selected behavioral factors in the early-life undernutrition exposed and unexposed cohorts of two community-based studies in Hua County, China, 2012-2023.

| **Variable** | **Total (N=8137)** |  | **Undernutrition exposed cohort^a^**  **(N=1948)** |  | **Unexposed cohort^b^**  **(N=6189)** | ***P* value^d^** |
| --- | --- | --- | --- | --- | --- | --- |
|  | **n (%)** |  | **n (%)** |  | **n (%)** |  |
| Age at enrollment ^c^(y) |  |  |  |  |  |  |
| Median (interquartile range) | 52 (50-54) |  | 55 (53-56) |  | 52 (50-53) | <0.001 |
| Sex |  |  |  |  |  |  |
| Female | 4447 (54.65) |  | 1044 (53.59) |  | 3403 (54.98) | 0.282 |
| Male | 3690 (45.35) |  | 904 (46.41) |  | 2786 (45.02) |  |
| Marital status |  |  |  |  |  |  |
| Others | 210 (2.58) |  | 58 (2.98) |  | 152 (2.46) | 0.206 |
| Married | 7927 (97.42) |  | 1890 (97.02) |  | 6037 (97.54) |  |
| Education |  |  |  |  |  |  |
| Illiterate | 1230 (15.12) |  | 440 (22.59) |  | 790 (12.76) | <0.001 |
| Primary School | 1500 (18.43) |  | 320 (16.43) |  | 1180 (19.07) |  |
| Junior High School | 3918 (48.15) |  | 736 (37.78) |  | 3182 (51.41) |  |
| High School and Above | 1489 (18.30) |  | 452 (23.20) |  | 1037 (16.76) |  |
| Occupation |  |  |  |  |  |  |
| Non-laborer | 237 (2.91) |  | 54 (2.77) |  | 183 (2.96) | 0.672 |
| Laborer | 7900 (97.09) |  | 1894 (97.23) |  | 6006 (97.04) |  |
| Cigarette smoking |  |  |  |  |  |  |
| No | 5510 (67.72) |  | 1292 (66.32) |  | 4218 (68.15) | 0.132 |
| Yes | 2627 (32.28) |  | 656 (33.68) |  | 1971 (31.85) |  |
| Alcohol drinking |  |  |  |  |  |  |
| No | 6198 (76.17) |  | 1492 (76.59) |  | 4706 (76.04) | 0.617 |
| Yes | 1939 (23.83) |  | 456 (23.41) |  | 1483 (23.96) |  |
| BMI (kg/m^2^) |  |  |  |  |  |  |
| <18.5 | 94 (1.16) |  | 24 (1.24) |  | 70 (1.13) | <0.001 |
| 18.5-24.9 | 3497 (42.98) |  | 925 (47.48) |  | 2572 (41.56) |  |
| 25.0-29.9 | 3585 (44.06) |  | 803 (41.22) |  | 2782 (44.95) |  |
| ≥30 | 961 (11.81) |  | 196 (10.06) |  | 765 (12.36) |  |
| Cancer family history |  |  |  |  |  |  |
| No | 5840 (71.77) |  | 1443 (74.08) |  | 4397 (71.05) | 0.010 |
| Yes | 2297 (28.23) |  | 505 (25.92) |  | 1792 (28.95) |  |

^a^ Early-life undernutrition exposed cohort consisted of participants born between 1 January 1959 and 31 December 1961, which was the duration of the Great Chinese Famine.

^b^ Unexposed cohort consisted of participants born between 1 January 1962 and 31 December 1964, which was after the Great Chinese Famine.

^c^ Participants in the ESECC trial were enrolled in this study in the period from January 2012 to September 2016, which was simultaneous with the baseline investigation.

^d^ *P* values were derived from the Chi-square test and Wilcoxon rank sum test.

**Supplemental table 4.** The mortality rate and cumulative mortality of all-cause death and chronic disease death in the early-life undernutrition exposed and unexposed cohorts with individual-level questionnaire data in Hua County, China, 2012-2023.

| **Outcome events** | **Undernutrition exposed cohorta (N=1948)** | | |  | **Unexposed cohortb (N=6189)** | | | ***P* value^c^** |
| --- | --- | --- | --- | --- | --- | --- | --- | --- |
|  | **Number of deaths** | **Mortality rate (per 1000 person-years)** | **Cumulative mortality (%)** |  | **Number of deaths** | **Mortality rate (per 1000 person-years)** | **Cumulative mortality (%)** |  |
| All-cause death | 72 | 4.23 (3.36-5.34) | 3.70 (2.90-4.63) |  | 148 | 2.71 (2.30-3.18) | 2.39 (2.03-2.80) | 0.002 |
| Cancer death | 27 | 1.59 (1.09-2.32) | 1.39 (0.92-2.01) |  | 47 | 0.86 (0.65-1.14) | 0.76 (0.56-1.01) | 0.011 |
| Cardiovascular and cerebrovascular diseases death | 33 | 1.94 (1.38-2.73) | 1.69 (1.17-2.37) |  | 55 | 1.01 (0.77-1.31) | 0.89 (0.67-1.16) | 0.003 |
| Chronic obstructive pulmonary disease death | 3 | 0.18 (0.06-0.55) | 0.15 (0.03-0.45) |  | 2 | 0.04 (0.01-0.15) | 0.03 (0.00-0.12) | 0.085 |

^a^ Early-life undernutrition exposure was defined as residents born between 1 January 1959 and 31 December 1961.

^b^ Non-exposure was defined as residents born between 1 January 1962 and 31 December 1964.

^c^ P values were derived from the Poisson regression.


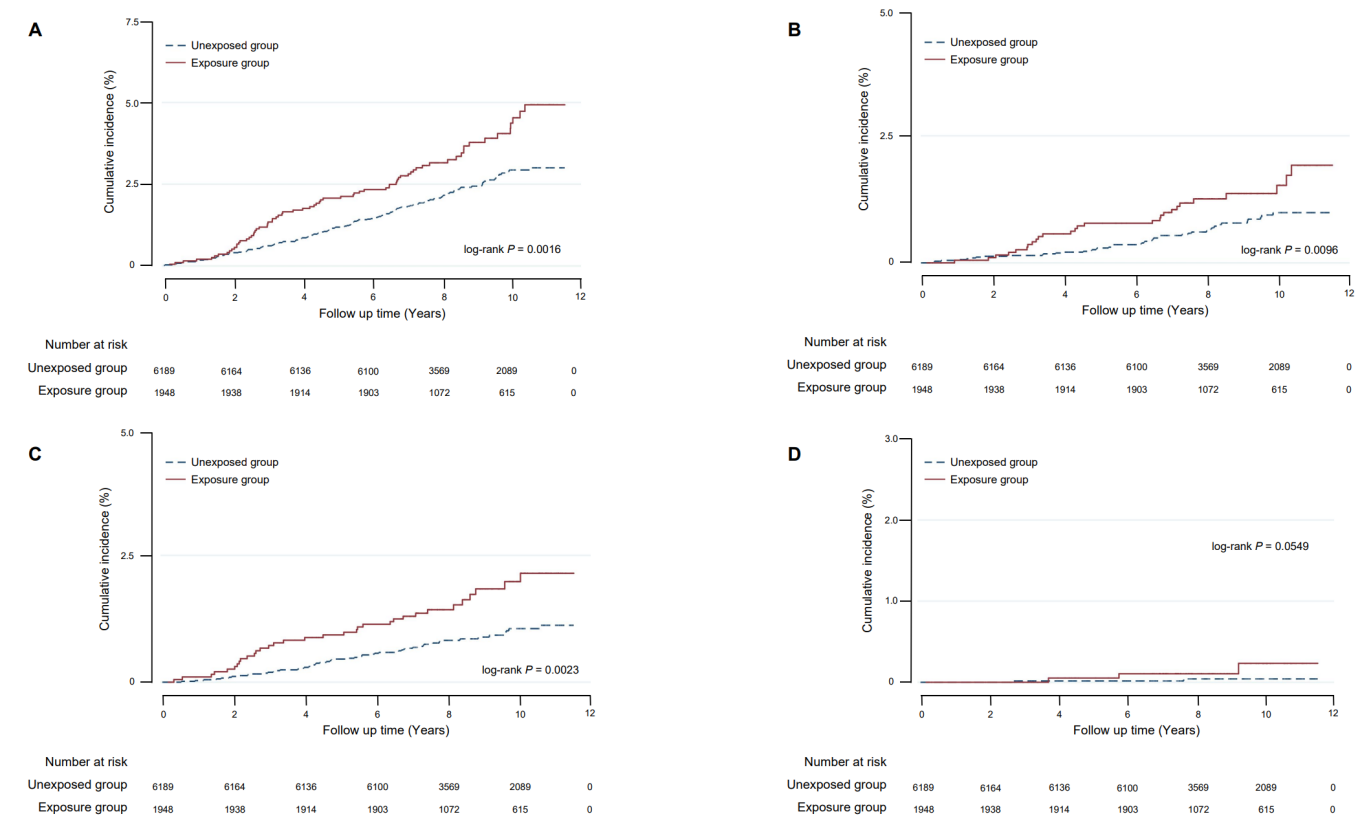


**Supplemental figure 1.** Cumulative incidence curves of all-cause death (A), cancer death (B), cardiovascular and cerebrovascular diseases death (C), and chronic obstructive pulmonary disease death (D) in early-life undernutrition exposed and unexposed group in the Endoscopic Screening for Esophageal Cancer in China (ESECC) trial and Anyang Esophageal Cancer Cohort Study (AECCS) in Hua County, China, 2012-2023.

Abbreviation: *HR:* hazard ratio; *CI*: confidence interval.

**Supplementary methods**

**Introduction to the ESECC trial**

The “Endoscopy Screening for Esophageal Cancer in China (ESECC)” trial was launched in 2012 in Hua County, Henan Province, a high-incidence area of esophageal cancer. This community-based trial (Clinical trials: NCT01688908) aims to evaluate the effect and cost-effectiveness of endoscopic screening.

1. Study Population

The study area of ESECC trial, Hua County, is located in the northern Taihang Mountains, with an esophageal cancer incidence rate of approximately 37 per 100,000. After excluding 112 villages with populations either too large (>3000) or too small (<500), 668 administrative villages with populations ranging from 500 to 3000 were randomly selected from 846 eligible villages using simple random numbers. These villages were then randomly assigned into screening or control arms using blocked cluster randomization according to the population size in each village (block size=2). Residents meeting the inclusion criteria in the target villages were enrolled voluntarily. The inclusion criteria were: (1) aged 45-69 years at the time of enrollment; (2) no history of cancer; (3) no contraindications to endoscopy (e.g., mental disorders, cardiovascular diseases, blood-borne diseases such as HBV/HCV/HIV); (4) having not received endoscopic examinations in the past five years. The baseline recruitment and examination of the ESECC trial were conducted during 2012-2016, with 17,151 and 16,797 eligible participants assigned to the screening and control groups, respectively. Ultimately, 15,299 participants in the screening group completed baseline upper gastrointestinal endoscopy and questionnaire surveys, while 16,764 participants in the control group completed baseline questionnaire surveys.

2. Screening Process

Participant enrollment and baseline screening were conducted on a village-by-village basis. The screening process for each participant included: (1) Registration: Eligible participants signed informed consent forms and provided valid identification for basic information registration, including name, sex, date of birth, address, contact information, and a frontal photograph. (2) Physical examination. (3) Blood collection. (4) Questionnaire survey: Participants completed a questionnaire at enrollment, covering age, sex, socioeconomic status (education level, household income per capita, occupation type), living environment, smoking, alcohol consumption, family history of esophageal cancer, main fuel type, kitchen smoke exposure, BMI, water source type, pesticide exposure, unhealthy dietary habits, self-reported upper digestive tract symptoms, personal disease history and cancer family history. (5) Upper gastrointestinal endoscopic examination with iodine staining in the screening arm, and the abdominal ultrasound examination in the control arm.

3. Follow-up

The ESECC cohort employed a combined approach of active and passive follow-up. Active follow-up involved annual door-to-door visits to obtain information on cancer incidence and all-cause mortality through interviews. Passive follow-up utilized data from Hua County’s “New Rural Cooperative Medical Scheme reimbursement system” and “death registration system” to track cancer incidence and all-cause death among participants. This combined approach ensures a follow-up coverage of over 99%, with high sensitivity and specificity in reporting incident cancer cases and death events.^1^

**Introduction to the AECCS study**

In 2006-2009, our research group conducted a population-based esophageal cancer cohort study in nine rural villages of Anyang, Henan Province, China, known as the Anyang Esophageal Cancer Cohort Study (AECCS). The study aims to investigate the prevalence of esophageal HPV infection in rural areas of northern Anyang, Henan Province, China. The primary endpoint is the detection of esophageal squamous cell carcinoma or precancerous lesions determined through endoscopic examination and pathological diagnosis, or clinically diagnosed esophageal squamous cell carcinoma.

1. Study Population

The AECCS study was conducted in five rural counties in the Hua county. Residents meeting the inclusion criteria in the target villages were enrolled voluntarily. The inclusion criteria were: (1) aged 25-65 years at the time of enrollment; (2) no history of malignant tumors; (3) no contraindications to endoscopy (e.g., mental disorders, severe cardiovascular diseases); (4) no history or evidence of hepatitis B virus (HBV), hepatitis C virus (HCV), or human immunodeficiency virus (HIV) infection.

During the baseline phase (2006-2009), a total of 11,554 permanent residents were assessed for eligibility, with 10,772 residents meeting the inclusion criteria after exclusions. Of these, 8,638 participants completed baseline interviews, and 8,112 underwent endoscopic examinations, resulting in a participation rate of 75.3%.

2. Screening Process

Over 8,000 residents met the inclusion criteria and were recruited through community announcements and door-to-door visits in the target villages. Each participant underwent a comprehensive baseline examination, including registration, screening for infectious diseases, detailed demographic and lifestyle questionnaires, collection of exfoliated cells, and chromoendoscopic examination with biopsy of the esophagus and stomach.

3. Follow-up

Follow-up examinations were conducted every two years. These included repeated questionnaire investigations and endoscopic examinations, focusing on changes in exposures of risk factors related to esophageal squamous cell carcinoma or HPV. Information on cancer incidence, all-cause death, and migrations were obtained through door-to-door visits, verified using local medical records and death certificates.

**Reference**

1 He Z, Liu Z, Liu M, et al. Efficacy of endoscopic screening for esophageal cancer in China (ESECC): design and preliminary results of a population-based randomised controlled trial. *Gut* 2019; **68:** 198-206.

2 Liu F, Guo F, Zhou Y, et al. The Anyang Esophageal Cancer Cohort Study: study design, implementation of fieldwork, and use of computer-aided survey system. *PloS one* 2012; **7:** e31602.

Flow diagram


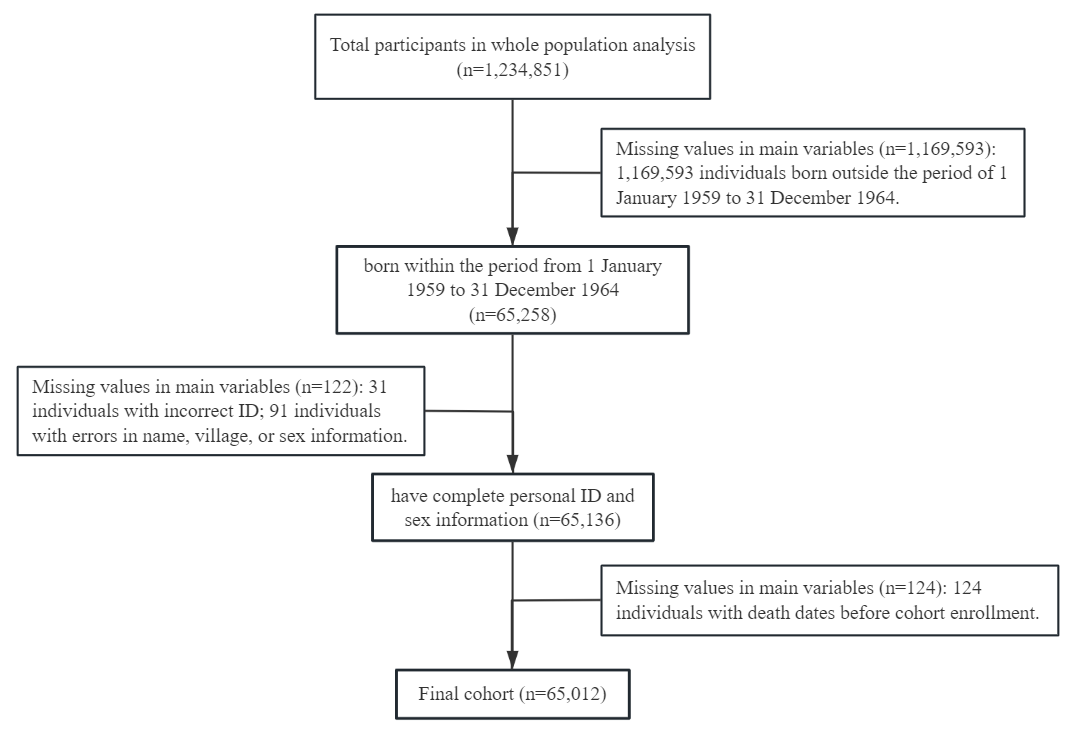

Supplement: Supplementary file 1 — Additional file 1. [file 41256_2025_422_MOESM1_ESM.docx]
